# Psychometric Properties of the Adult Executive Functioning Inventory: Application of Rasch Analyses

**DOI:** 10.1002/brb3.71453

**Published:** 2026-04-27

**Authors:** Chia‐Wei Fan, Pai‐Cheng Lin, Cheng‐Fang Yen, Chung‐Ying Lin

**Affiliations:** ^1^ Department of Occupational Therapy AdventHealth University Orlando Florida USA; ^2^ Department of Psychiatry Kaohsiung Medical University Gangshan Hospital Kaohsiung Taiwan; ^3^ Department of Psychiatry Kaohsiung Medical University Hospital Kaohsiung Taiwan; ^4^ Department of Psychiatry, School of Medicine, College of Medicine Kaohsiung Medical University Kaohsiung Taiwan; ^5^ Institute of Allied Health Sciences, College of Medicine National Cheng Kung University Tainan Taiwan; ^6^ Biostatistics Consulting Center, National Cheng Kung University Hospital, College of Medicine National Cheng Kung University Tainan Taiwan; ^7^ Department of Public Health, College of Medicine National Cheng Kung University Tainan Taiwan; ^8^ Department of Occupational Therapy, College of Medicine National Cheng Kung University Tainan Taiwan

**Keywords:** adult executive functioning inventory, flexibility, inhibition, psychometric properties, Rasch analyses, working memory

## Abstract

**Introduction:**

Executive function is important ability for individuals to deal with daily living activities, and the Adult Executive Functioning Inventory (ADEXI) with promising psychometric properties was developed to assess executive function in nonclinical adult populations. However, psychometric properties evidence for ADEXI was primarily based on classical test theory; therefore, the current study used Rasch analysis to examine ADEXI to provide additional psychometric properties evidence.

**Methods:**

Participants aged 18–25 years (*n* = 1764; 55.9% women) were recruited using a social media platform (*Dcard*). They completed self‐report Mandarin version of the ADEXI (of which includes three subscales of working memory, inhibition, and flexibility) and demographic characteristics (age, gender, and educational level) via online survey. Rasch analyses with differential item functioning (DIF) were used as analytic methods.

**Results:**

All ADEXI items aligned with their intended subscales, with fit statistics of mean square ranging between 0.5 and 1.5. Moreover, the 5‐point Likert scale of the ADEXI followed its order to indicate the difficulty (i.e., a higher score in the Likert scale indicates a poorer level of executive function). Moreover, no DIF items were displayed for the ADEXI across gender subgroup (men vs. women) or educational subgroup (college or above vs. high school or below).

**Conclusion:**

The psychometric properties of ADEXI were satisfactory, as indicated by the Rasch analysis findings. Unidimensionality of each ADEXI subscale was supported, categorical function was in order for the response, and no items displayed DIF. Therefore, the ADEXI can be used to evaluate executive function for general population and to enhance health promotion.

## Graphical abstract

Adult Executive Functioning Inventory (ADEXI) assesses executive function in nonclinical adult population in three domains: working memory, inhibition, and flexibility. The present study extended the psychometric properties of ADEXI from traditional classical test theory to Rasch analysis. Specifically, each domain is unidimensional, and all ADEXI items are interpreted similarly across gender groups and across educational level groups.

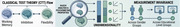

## 1 Introduction

Executive function refers to a complex set of higher order cognitive operations that includes working memory, inhibitory control, cognitive flexibility, planning, reasoning, and problem solving (Cristofori et al. 2019; Lezak et al. 2012; Welsh and Pennington 1988). Executive function controls and regulates a person's thoughts, emotions, and behaviors (Lezak et al. 2012). Consequently, deficits in executive function have dramatic impacts on everyday life, which include poor goal‐directed behavior, impaired social functioning, and mental and physical health problems (Cristofori et al. 2019; Salehinejad et al. 2021). Low executive function can contribute to diminished quality of life and a proclivity toward crime and violence (Hancock et al. 2010; Siman‐Tov et al. 2016).

Executive function deficits have been found in several psychiatric disorders, such as attention‐deficit/hyperactivity disorder (ADHD), autism spectrum disorders, anxiety disorders, bipolar disorders, depressive disorders, schizophrenia, substance use disorders, internet gaming disorder, sleep disorders, and eating disorders (Ballesio et al. 2019; Baroni et al. 2025; Cesari et al. 2021; Diaz‐Marsa et al. 2023; Lima et al. 2018; Salehinejad et al. 2021; Townes et al. 2023). A meta‐analysis demonstrated that moderate deficits in executive function are associated with having depressive disorder; the significant association persists in individuals whose depressive symptoms had remitted (Rock et al. 2014). Anxiety disorders have also been found to be bidirectionally correlated with deficits in executive function (Zainal and Newman 2022). Further, deficits in executive function have been identified as a core impairment in ADHD in children (Groves et al. 2022) and adults (Barkley 2010).

Researchers have developed several self‐report scales for measuring the level of executive function in adults, such as the Behavior Rating Inventory of Executive Functions–Adult version (Roth et al. 2005), Dysexecutive Questionnaire (DQ) (Wilson et al. 1996), and Barkley's Deficits in Executive Function Scale (BDEFS) (Barkley 2011). However, these instruments especially focus on clinical populations and thus may have somewhat low levels of applicability in nonclinical populations. Specifically, a section of these measurement scales is dedicated to assessing symptoms of specific psychiatric disorders (e.g., ADHD). Thus, these scales contain numerous items, which are difficult to apply to nonclinical populations at scale (Holst and Thorell 2018). Moreover, some items contained in these scales do not directly measure executive function (Holst and Thorell 2018). In this regard, a measure particularly designed for nonclinical populations to assess executive function is needed.

The Adult Executive Functioning Inventory (ADEXI) was developed for the assessment of executive function in nonclinical adult populations (Holst and Thorell 2018). The ADEXI was developed according to Baddeley and Hitch (1974) and Barkley (1997). Baddeley and Hitch (1974) emphasized that working memory is comprised of multiple components involved in the storage of verbal and spatial information, as well as the processing of such information, which are required when performing multiple tasks simultaneously, or in tasks that involve several steps. Barkley's (1997) hybrid model emphasized that inhibition, working memory, and flexible regulation are commonly seen as constituting the major EF deficits in individuals with psychiatric disorders such as ADHD. Studies have demonstrated the ADEXI's high internal consistency and adequate test–retest reliability (Holst and Thorell 2018) and have revealed significant correlations between ADEXI score and scores on other instruments measuring executive function, such as the DQ and BDEFS (Fogel et al. 2024; García‐Villamisar et al. 2020; Holst and Thorell 2018). ADEXI score was also significantly correlated with problematic cannabis and alcohol use and problematic gambling (Baroni et al. 2025). The ADEXI has been translated into several languages and has been widely demonstrated to be valid (Baroni et al. 2025; Fogel et al. 2024; García‐Villamisar et al. 2020).

Although prior ADEXI studies (Baroni et al. 2025; Fogel et al. 2024; García‐Villamisar et al. 2020; Holst and Thorell 2018) have provided psychometric evidence to support the application of ADEXI, they largely relied on classical test theory. Such psychometric evidence from classical test theory does not fully evaluate how individual items perform along the executive functioning continuum, or whether raw ordinal totals can be interpreted as a linear measure. In contrast, Rasch measurement addresses these gaps by testing whether ADEXI responses conform to model expectations for constructing a unidimensional, interval‐level latent variable, while simultaneously evaluating item fit, person and item separation, and how well item locations are targeted to the sample (Bond and Fox 2007; Tennant and Conaghan 2007).

Because the ADEXI uses Liker‐style responses (i.e., response options in categories), Rasch analysis outperforms psychometric evaluation methods using classical test theory to directly examine category threshold ordering, and whether response options function as intended, with guidance from the established category‐functioning criteria (Andrich 1978; Linacre 2002a). Moreover, Rasch‐based differential item functioning (DIF) analyses provide a rigorous test of measurement invariance, clarifying whether items operate equivalently across demographic subgroups and supporting fair comparisons of scores (Dorans and Holland 1993; Tennant and Conaghan 2007).

In order to fill up the psychometric evidence of ADEXI, the present study aimed to examine the psychometric properties of the ADEXI using Rasch measurement. Specifically, we (1) evaluated the functioning of the 5‐point Likert responses to determine whether category thresholds and adjacent categories advanced monotonically as intended; (2) examined item fit to Rasch model expectations and residual dimensionality as evidence supporting ADEXI's construct validity; (3) examined the person and item separation indices to assess the scale's ability to distinguish among respondents and item difficulty levels; (4) tested measurement invariance by evaluating DIF across gender and educational level; and (5) assessed floor and ceiling effects for each ADEXI subscale.

## 2 Methods

### 2.1 Study Cohort and Procedure

This survey study posted advertisements on *Dcard* once every week between August and October 2025 to recruit young adults for an online survey. *Dcard* is a social media platform widely used by young adults in Taiwan and has more than 10 million users. People living in Taiwan aged 18–25 years were eligible for inclusion. This study had no exclusion criteria. The advertisement provided details on the study's objectives, instructions for completing the online questionnaire, and assurances regarding data confidentiality and participant privacy. Interested individuals could access the questionnaire by clicking on the “Agree to Participate” button, after which they proceeded to provide their responses. Those who did not wish to participate could either opt out by selecting the “Not Willing to Participate” button or by simply disregarding the advertisement. In total, 1764 individuals completed the online survey. The study protocol was approved by the Institutional Review Board of Kaohsiung Medical University Hospital (KMUHIRB‐E(I)‐20240422).

### 2.2 Measures

#### 2.2.1 Adult Executive Functioning Inventory

Executive functioning was evaluated using the self‐report Mandarin version of the ADEXI, which we translated from its original English version (Holst and Thorell 2018). We conducted this translation through a standardized forward–backward translation process (Sousa and Rojjanasrirat 2011). The ADEXI comprises 14 items (e.g., “*When someone asks me to do several things, I sometimes remember only the first or last*”) scored on a 5‐point Likert scale ranging from 1 (*definitely not true*) to 5 (*definitely true*) across three subscales: working memory, inhibition, and flexibility. A higher score on a subscale indicates a greater deficit in executive functioning.

#### 2.2.2 Demographic Characteristics

Participants’ age, gender (man vs. woman), and educational level (college or above vs. high school or below) were collected.

### 2.3 Data Analysis

Rasch analyses were conducted using *Winsteps* software (version 5.10.2.0) and *Facets* (version 4.4.1) to evaluate DIF and other psychometric properties of the ADEXI. Descriptive and demographic statistics were computed using *IBM SPSS Statistics* *Software* (version 31.0). Participants’ demographic characteristics were summarized using means and standard deviations for ratio‐level variables (i.e., age) and frequencies with percentages for categorical variables (i.e., gender and educational level).

A recent factor analytic study has confirmed the three‐factor structure of the ADEXI, consisting of working memory, inhibition, and flexibility subscales (Fogel et al. 2024). We also conducted competing models to compare the two‐factor structure and three‐factor structure of ADEXI among Taiwanese young adults, and the results indicated that the three‐factor structure performed significantly better than two‐factor structure (Δ_scaled_ *χ* ^2^ = 7.548; Δ_df_ = 2; *p* = 0.023). Therefore, the present study decided to use the three‐factor structure to test ADEXI. In the current analyses, raw scores for each subscale were summed and analyzed separately. Rasch analysis was applied to transform raw scores into interval‐level measures, providing estimates of participants’ executive functioning in the three subscales. Within the Rasch framework, person ability Rasch estimates represent participants’ levels of executive functioning, whereas item difficulty Rasch calibrations indicate the level of executive functioning required for endorsement of each item along the latent trait continuum.

DIF was evaluated by comparing item calibrations across two subgroup categories: gender (man vs. woman) and educational level (college or above vs. high school or below). Item difficulty estimates expressed in logits were calculated separately for each subgroup and used to compute DIF contrasts. A DIF contrast exceeding an absolute value of 0.64 logits was used to flag items exhibiting both statistically significant DIF and a meaningful effect size, with values above this threshold indicating moderate to large DIF (Zwick et al. 1999).

The partial credit model (Masters 2019) was employed for the Rasch analyses of the three ADEXI subscales. Response‐category functioning was evaluated using average category measures, category threshold estimates, and the frequency distribution of category use to determine whether the five response options functioned as intended. Item fit was evaluated using goodness‐of‐fit statistics derived from the Rasch analysis to assess unidimensionality and support construct validity. Mean square (MnSq) and standardized *z* values (Zstd) were used as indicators of model‐data fit. Infit MnSq values close to 1.0 indicate good model fit, values below 1.0 suggest overly predictable responses, and values above 1.0 indicate unmodeled noise or unexpected response patterns. In accordance with established guidelines, acceptable item fit was defined as Infit MnSq values between 0.5 and 1.5, accompanied by standardized *z* values (Zstd) between −2 and +2 (Linacre 2002b). When MnSq values fell within the acceptable range, Zstd values were not interpreted further. Residual dimensionality was further examined using principal components analysis (PCA) of standardized residuals with the criterion that the eigenvalue of the first contrast was less than 3. Additionally, Rasch modeled variances were expected to be close to the empirical variance (Linacre 2026). Person and item separation indices and associated reliability coefficients were calculated to evaluate the ability of each subscale to distinguish among participants and the stability of item calibration estimates. Targeting was assessed by comparing the mean person measure with the item mean fixed at 0 logits for each ADEXI subscale.

Ceiling and floor effects were examined by evaluating the proportion of participants who achieved the highest or lowest possible scores on each ADEXI subscale. Consistent with prior recommendations, ceiling or floor effects were considered present if more than 15% of participants attained the maximum or minimum possible score, respectively (Gulledge et al. 2020).

## 3 Results

### 3.1 Participants’ Characteristics

A total of 1764 participants from a higher education population were included in the study. The mean age of the sample was 22.3 years (SD = 1.76). Women participants comprised 55.9% of the sample, whereas 44.1% identified as men. For the educational level, 86.62% of participants reported having a college education or above, and 12.38% reported a high school education or below. Additional demographic details are presented in Table 1.

**TABLE 1:** Demographics (*N* = 1764).

Variables	*M* (SD)/*n* (%)
**Age**	22.3 (1.76)
**Gender**	
Male	778 (44.10)
Female	986 (55.90)
**Educational level**	
College or above	1528 (86.62)
High school or below	236 (12.38)

Abbreviation: SD, standard deviation.

### 3.2 Rating Scale Functioning, Rasch Model Fit, Person/Item Separation, and Ceiling and Floor Effects

Across the three ADEXI subscales, the 5‐point Likert response categories demonstrated monotonically increasing average rating measures, indicating appropriate functioning of the rating scale (Table 2). In addition, the Andrich thresholds were ordered across items within each subscale. For the *working memory* subscale, the first through fourth step thresholds ranged from −4.75 to −3.73, −1.24 to −0.79, 1.53 to 1.77, and 3.08 to 4.11 logits, respectively. Corresponding ranges for the *inhibition* subscale were −2.55 to −2.10, −1.12 to −0.61, 0.66 to 1.19, and 2.17 to 2.50 logits, and for the *flexibility* subscale, they were −5.09 to −4.87, −1.49 to −1.02, 1.84 to 1.95, and 3.99 to 4.58 logits. These findings supported the ordered functioning of the five response categories across the three ADEXI subscales. The frequency distributions of category use for each item across the five response options are reported in Table 2.

**TABLE 2:** Rasch analysis results of the 14 Adult Executive Functioning Inventory (ADEXI) items.

Item	Measure	SE	Infit		Outfit		Average rating measure^a^/Data count				
			*MnSq*	*Zstd*	*MnSq*	*Zstd*	1	2	3	4	5
**Working memory**											
1	−0.40	0.04	0.96	−1.07	0.96	−1.21	−5.27/176	−2.27/601	−0.39/675	1.28/244	2.85/68
2	0.24	0.04	0.86	−4.24	0.86	−4.13	−4.91/224	−1.97/693	−0.08/613	1.68/201	3.86/33
5	0.08	0.04	0.83	−5.33	0.83	−5.02	−4.61/268	−2.13/583	−0.06/651	1.48/212	3.14/50
8	−0.22	0.04	0.99	−0.37	0.99	−0.39	−5.50/172	−2.04/691	−0.22/615	1.18/233	3.34/53
9	0.31	0.04	1.33	8.74	1.30	7.77	−3.89/331	−1.71/666	0.03/539	1.15/172	2.95/56
**Inhibition**											
3	0.16	0.03	0.82	−5.73	0.83	−5.61	−2.08/257	−1.04/529	−0.07/608	0.67/304	1.74/66
4	0.02	0.03	0.77	−7.58	0.77	−7.56	−2.32/208	−1.16/461	−0.18/724	0.72/302	1.71/69
6	0.63	0.03	0.85	−4.91	0.83	−5.37	−1.91/333	−0.70/691	0.05/500	1.00/204	2.34/36
10	−1.01	0.03	1.04	1.27	1.06	1.80	−2.78/89	−1.60/239	−0.63/690	0.14/500	0.76/246
14	0.20	0.03	1.48	9.90	1.51	9.90	−1.47/233	−0.80/569	−0.30/684	0.62/217	1.05/61
**Flexibility**											
7	−0.30	0.04	1.08	2.29	1.08	2.25	−5.57/191	−2.46/627	−0.17/641	1.35/243	3.15/62
11	−0.35	0.04	1.07	2.08	1.07	1.92	−5.65/171	−2.77/546	−0.32/738	1.44/264	3.14/45
12	0.31	0.05	0.85	−4.35	0.82	−5.20	−5.28/228	−2.25/714	0.19/611	2.09/179	4.06/32
13	0.34	0.04	0.94	−1.62	0.95	−1.43	−5.10/240	−2.18/727	0.21/584	2.02/180	3.78/33

Abbreviations: MnSq, mean square; SE, standard error; Zstd, standardized score.

^a^ The average measure is expected to increase with category value (Linacre, 2002a).

^+^Infit statistics >1.5 associated with Zstd >2: item misfit.

The item fit statistics derived from the Rasch analysis supported the unidimensionality of each subscale. Infit MnSq values for all items fell within the predefined acceptable range, supporting adequate model fit and construct validity. Detailed item‐level fit statistics and Rasch calibration estimates are reported in Table 2. PCA of standardized residuals further supported the essential unidimensionality of the three ADEXI subscales. For the *working memory* subscale, the raw variance explained by the Rasch measures was 58.0%, which closely matched the model‐expected variance of 58.1%, and the eigenvalue of the first residual contrast was 1.70. For the *inhibition* subscale, the raw variance explained by the Rasch measures was 49.3% (expected = 48.8%), and the eigenvalue of the first residual contrast was 1.50. For the *flexibility* subscale, the raw variance explained by the Rasch measures was 62.6% (expected = 62.3%), and the eigenvalue of the first residual contrast was 1.53. Because all first‐contrast eigenvalues were below the prespecified criterion of 3.0 and the observed variances explained by the Rasch measures were close to the expected values, the PCA results indicated no substantial secondary dimension and supported the unidimensional structure of each ADEXI subscale.

Moderate person separation/reliability was found 2.20/0.83 for *working memory*, 1.50/0.69 for *inhibition*, and 2.09/0.81 for *flexibility*. In contrast, item separation/reliability was high across all three subscales, at 6.30/0.98, 16.07/1.00, and 7.12/0.98, respectively, indicating stable item difficulty estimates. Regarding person‐item targeting, the mean person measures for the full sample were −1.16 logits for *working memory*, −0.46 logits for *inhibition*, and −1.24 logits for *flexibility* relative to the item mean fixed at 0 logits. This pattern suggests that the *inhibition* subscale was more closely targeted to the sample, whereas the *working memory* and *flexibility* subscales were somewhat more difficult to endorse in this sample.

An examination of response distributions revealed minimal ceiling and floor effects. Specifically, nine participants (0.51%), eight participants (0.45%), and seven participants (0.40%) achieved the highest possible scores on the *working memory*, *inhibition*, and *flexibility* subscales, respectively. Conversely, 92 participants (5.21%), 14 participants (0.79%), and 66 participants (3.74%) achieved the lowest possible scores on the corresponding subscales. As these proportions were well below the 15% criterion, no ceiling or floor effects were observed for any ADEXI subscale.

### 3.3 DIF by Gender

**FIGURE 1:**
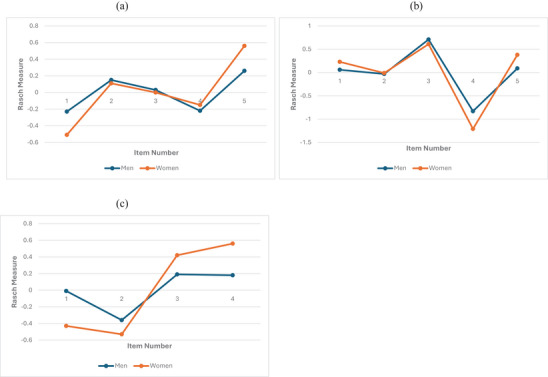
**Differential item functioning across gender**. Items are listed in sequential order for analysis. The working memory subscale includes ADEXI items 1, 2, 5, 8, and 9; the inhibition subscale includes items 3, 4, 6, 10, and 14; and the flexibility subscale includes items 7, 11, 12, and 13. (a) Working memory subscale; (b) inhibition subscale; (c) flexibility subscale.

DIF analyses indicated no meaningful DIF across gender groups. The absolute DIF contrast values ranged from 0.02 to 0.42 logits across the three subscales (Table 3). Six ADEXI items (Items 1, 7, 9, 10, 13, and 14) exhibited statistically significant DIF at the *p* < 0.01 level. However, all statistically significant DIF contrasts remained below the 0.64‐logit threshold, indicating that none of these items demonstrated meaningful DIF. Visual inspections of the Rasch logit line plots further supported these findings, with parallel item difficulty estimates observed across male and female participants for all three subscales. Please see Figure 1.

**TABLE 3:** Gender differential item functioning (DIF) for the 14 Adult Executive Functioning Inventory (ADEXI) items.

Item	Calibration		*t*	*p*	DIF contrast^a^
	Male	Female			
**Working memory**					
1. I have difficulty remembering lengthy instructions	−0.23	−0.51	3.42	0.00	0.28
2. I sometimes have difficulty remembering what I am doing in the middle of an activity	0.15	0.11	0.46	0.65	0.04
5. When someone asks me to do several things, I sometimes remember only the first or last	0.03	0.00	0.40	0.69	0.03
8. When someone asks me to fetch something, I sometimes forget what I am supposed to fetch	−0.22	−0.15	−0.88	0.38	−0.07
9. I have difficulty planning for an activity	0.26	0.56	−3.47	0.00	−0.29
**Inhibition**					
3. I have a tendency to do things without first thinking about what could happen	0.06	0.23	−2.56	0.01	−0.17
4. I sometimes have difficulty stopping myself from doing things that I like even though someone tells me that it is not allowed	−0.03	−0.01	−0.30	0.76	−0.02
6. I sometimes have difficulty refraining from smiling or laughing in situations where it is inappropriate	0.71	0.61	1.43	0.15	0.09
10. I sometimes have difficulty stopping an activity that I like	−0.83	−1.21	5.85	0.00	0.38
14. People that I meet sometimes seem to think that I am livelier/wilder compared with other people my age	0.09	0.38	−4.46	0.00	−0.29
**Flexibility**					
7. I have difficulty coming up with a different way of solving a problem when I get stuck	−0.01	−0.43	4.69	0.00	0.42
11. I sometimes have difficulty understanding verbal instructions unless I am also shown how to do something	−0.36	−0.53	1.89	0.06	0.17
12. I have difficulties with tasks or activities that involve several steps	0.19	0.42	−2.59	0.01	−0.23
13. I have difficulty thinking ahead or learning from experience	0.18	0.56	−4.14	0.00	−0.38

Abbreviation: DIF, differential item functioning.

^a^ Negative values indicate that the probability of the item endorsement is easier for male participants than female participants.

### 3.4 DIF by Educational Level

**FIGURE 2:**
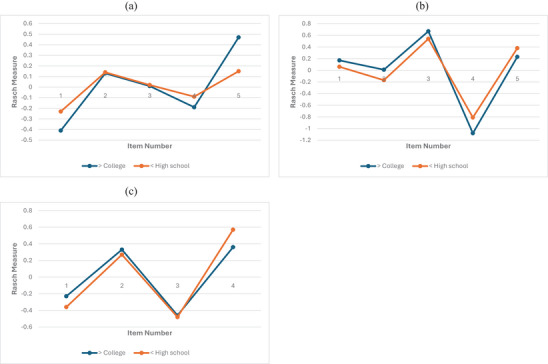
**Differential item functioning across educational levels**. Items are listed in sequential order for analysis. The working memory subscale includes ADEXI items 1, 2, 5, 8, and 9; the inhibition subscale includes items 3, 4, 6, 10, and 14; and the flexibility subscale includes items 7, 11, 12, and 13. (a) Working memory subscale; (b) inhibition subscale; (c) flexibility subscale.

Similarly, no meaningful DIF was detected across educational level groups. Absolute DIF contrast values ranged from 0.01 to 0.32 logits across the *working memory*, *inhibition*, and *flexibility* subscales (Table 4). One item (Item 10) demonstrated statistically significant DIF at the *p* < 0.01 level; however, the magnitude of the DIF contrast did not exceed the 0.64‐logit criterion and therefore was not considered meaningful. Figure 2 presents line plots of Rasch item difficulties across educational levels in the three subscales, illustrating the consistency of item functioning across groups.

**TABLE 4:** Education differential item functioning (DIF) for the 14 Adult Executive Functioning Inventory (ADEXI) items.

Item	Calibration		*t*	*p*	DIF contrast^a^
	College and above	High school and below			
**Working memory**					
1. I have difficulty remembering lengthy instructions	−0.41	−0.23	−1.56	0.12	−0.19
2. I sometimes have difficulty remembering what I am doing in the middle of an activity	0.13	0.14	−0.08	0.94	−0.01
5. When someone asks me to do several things, I sometimes remember only the first or last	0.01	0.02	−0.11	0.91	−0.01
8. When someone asks me to fetch something, I sometimes forget what I am supposed to fetch	−0.19	−0.09	−0.86	0.39	−0.10
9. I have difficulty planning for an activity	0.47	0.15	2.66	0.01	0.32
**Inhibition**					
3. I have a tendency to do things without first thinking about what could happen	0.17	0.06	1.24	0.22	0.12
4. I sometimes have difficulty stopping myself from doing things that I like even though someone tells me that it is not allowed	0.01	−0.17	1.94	0.05	0.18
6. I sometimes have difficulty refraining from smiling or laughing in situations where it is inappropriate	0.67	0.54	1.41	0.16	0.13
10. I sometimes have difficulty stopping an activity that I like	−1.08	−0.81	−2.92	0.00	−0.27
14. People that I meet sometimes seem to think that I am livelier/wilder compared with other people my age	0.23	0.38	−1.63	0.10	−0.15
**Flexibility**					
7. I have difficulty coming up with a different way of solving a problem when I get stuck	−0.23	−0.36	0.97	0.34	0.12
11. I sometimes have difficulty understanding verbal instructions unless I am also shown how to do something	0.33	0.27	0.40	0.69	0.05
12. I have difficulties with tasks or activities that involve several steps	−0.46	−0.48	0.20	0.84	0.03
13. I have difficulty thinking ahead or learning from experience	0.36	0.57	−1.59	0.11	−0.21

Abbreviation: DIF, differential item functioning.

^a^ Negative values indicate that the probability of the item endorsement is easier for college and above participants than high school and below participants.

## 4 Discussion

The current study used Rasch analyses to extend psychometric evidence of ADEXI from prior research (Baroni et al. 2025; Fogel et al. 2024; García‐Villamisar et al. 2020; Holst and Thorell 2018). In particular, prior research primarily reports psychometric evidence utilizing classical test theory; therefore, several properties of ADEXI (e.g., ordering of the response scale and DIF across subgroups) remained understudied. To the best of the authors’ knowledge, this study is the first to use Rasch analyses on ADEXI and to provide the following psychometric evidence: The five‐category response format of the ADEXI functioned as intended, with monotonically advancing average measures and ordered Andrich thresholds across the three subscales. All items demonstrated acceptable Rasch fit, and PCA of standardized residuals supported the essential unidimensionality of the working memory, inhibition, and flexibility subscales. Person separation was moderate, whereas item separation was high across subscales, indicating adequate differentiation among respondents and stable item difficulty estimates. In addition, the absence of floor or ceiling effects suggests that these subscales capture meaningful variability in executive functioning deficits in the young adult community population. DIF analyses further indicated that item calibrations were practically invariant across gender and educational levels, supporting fair group comparisons.

Beyond supporting overall model fit, the Rasch calibrations provide an interpretable hierarchy of item endorsement within each ADEXI subscale in this nonclinical sample of young adults. For example, in the *working memory* subscale, endorsing difficulty remembering lengthy instructions (Item 1; −0.40 logits) and forgetting what one was supposed to fetch (Item 8; −0.22 logits) reflected relatively milder problems that were more readily endorsed in this nonclinical sample. In contrast, difficulty remembering what one is doing in the middle of an activity (Item 2; 0.24 logits) and difficulty planning for an activity (Item 9; 0.31 logits) were the most “difficult” items to endorse, suggesting that these items may reflect comparatively higher levels of working‐memory‐related executive dysfunction. This pattern aligns with prior evidence that minor lapses in attention and memory are common in everyday life among healthy individuals (Carrigan and Barkus 2016). In contrast, difficulties that disrupt ongoing activities or interfere with planning and carrying out intended actions may reflect more consequential executive problems, because such failures are typically experienced as more serious and can have greater day‐to‐day impact (Niedźwieńska et al. 2020). Thus, occasional instruction‐related slips may represent commonplace cognitive failures, whereas interruptions in ongoing tasks and planning‐related breakdowns may signal broader difficulties with functional implications for academic, work, and independent living demands (Couffe and Michael 2017).

Within the *inhibition* subscale, difficulty stopping an enjoyable activity (Item 10; −1.01 logits) was most easily endorsed, consistent with a common, everyday self‐regulation challenge. By contrast, difficulty refraining from smiling or laughing in inappropriate situations (Item 6; 0.63 logits) was the most difficult to endorse, which may reflect a more overt and socially salient form of behavioral disinhibition. For *flexibility* subscale, items reflecting difficulty understanding verbal instructions without demonstration (Item 11; −0.35 logits) and difficulty generating an alternative solution when stuck (Item 7; −0.30 logits) were easier to endorse, whereas difficulties with multistep activities (Item 12; 0.31 logits) and thinking ahead or learning from experience (Item 13; 0.34 logits) were harder to endorse and may indicate higher levels of executive functional impact. These item hierarchies derived from Rasch analysis should be interpreted cautiously. Because the present study was conducted in a nonclinical sample, the Rasch difficulty ordering is better understood as reflecting the relative endorsement of executive‐function‐related experiences in community‐dwelling young adults rather than a direct index of clinical severity. Accordingly, the hierarchy provides a useful descriptive interpretation of how different executive difficulties were distributed in the enrolled sample, but further research in clinical populations is needed before drawing stronger conclusions about their clinical significance.

The DIF findings provide additional evidence that the ADEXI items function comparably across key demographic groups in this sample. Gender DIF contrasts were uniformly small, with absolute values up to 0.42 logits, well below the a priori criterion for meaningful DIF. Educational‐level DIF results were similarly reassuring, with absolute contrasts up to 0.32 logits. These results suggest that any observed group differences in subscale scores are unlikely to reflect systematic item bias, and that ADEXI scores are comparable between men and women and between participants with college education or above versus high school or below. Visual inspection of the item‐difficulty line plots further corroborated these findings, showing closely aligned item locations across groups and supporting measurement invariance. Overall, the minimal DIF across both gender and education supports using the ADEXI subscales for fair group comparisons in Taiwanese young adults without the need for group‐specific scoring or item adjustments (Martinková et al. 2017).

Score distributions indicated that the ADEXI subscales were not affected by extreme‐score clustering in this sample. Ceiling scores were rare across the *working memory*, *inhibition*, and *flexibility* subscales (0.40%–0.51%), indicating that the measures captured variability in executive dysfunction without saturating at the upper end. Floor scores were also well below the conventional 15% criterion (0.79%–5.21%), suggesting adequate discrimination at the lower end of the scale. Overall, these findings support the sensitivity of the ADEXI subscales for detecting individual differences in executive functioning deficits among community‐dwelling young adults.

The results of this study supported the usage of the ADEXI for assessing executive functioning in non‐clinical populations; its potential role in clinical screening and applied settings warrants further study. However, several limitations should be considered when interpreting these findings. First, participants were recruited through *Dcard* using a voluntary online survey, which may introduce self‐selection and limit generalizability beyond Taiwanese young adults who use social media. Second, the sample was restricted to ages 18–25 years, which may limit the applicability of these results to older adult populations. Third, although the high school or below subgroup exceeded 200 participants, the education groups were substantially imbalanced in sample sizes, and unequal group sizes can affect the performance and precision of DIF detection; thus, education‐related DIF estimates for the smaller subgroup should be interpreted with appropriate caution (Herrera and Gómez 2008). Fourth, in the current study, executive functioning was self‐reported, which is susceptible to social desirability and may not fully align with performance‐based executive function tasks (Latkin et al. 2017). Future studies should therefore replicate these Rasch findings in more diverse samples spanning broader age ranges, educational levels, and clinical groups. It would also be useful to examine convergent and known‐groups validity by linking ADEXI measures to performance‐based executive function tasks and to relevant clinical indicators, as well as to assess longitudinal responsiveness to determine whether the ADEXI can sensitively track changes over time.

## 5 Conclusion

In conclusion, the present study examined the psychometric properties of ADEXI via Rasch analysis. The findings indicated that each subscale of ADEXI is unidimensional with ordered category functions (i.e., a higher score in the Likert scale response indicates a poorer level of executive function). Moreover, no substantial DIF was displayed for all 14 ADEXI items across either gender subgroup or educational level subgroup, indicating that all the ADEXI items have no bias in comparing executive functions across different genders or different educational levels. Therefore, the ADEXI can be used to evaluate executive function for general population to enhance health promotion.

## Author Contributions

**Chia‐Wei Fan**: investigation, methodology, validation, writing – original draft, writing – review and editing, formal analysis, visualization, software. **Pai‐Cheng Lin**: data curation, project administration, validation, writing – review and editing, funding acquistion, investigation. **Cheng‐Fang Yen**: data curation, supervision, project administration, methodology, writing – review and editing, writing – original draft, conceptualization, investigation, funding acquisition. **Chung‐Ying Lin**: conceptualization, validation, methodology, writing – original draft, writing – review and editing, supervision.

## Funding

This work was partially supported by grants from the Kaohsiung Medical University Gangshan Hospital (KMUGH‐114‐043).

## Ethics Statement

The study protocol was approved by the Institutional Review Board of Kaohsiung Medical University Hospital (KMUHIRB‐E(I)‐20240422).

## Consent

Individuals interested in participating were able to access the questionnaire by clicking an “Agree to Participate” button, after which they proceeded to provide their responses. Those who did not wish to participate could opt out by selecting a “Not Willing to Participate” button or by simply ignoring the advertisement.

## Conflicts of Interest

The authors declare no conflicts of interest.

## Data Availability

Data will be made available on reasonable request.

## References

[brb371453-bib-0001] Andrich, D. 1978. “A Rating Formulation for Ordered Response Categories.” Psychometrika 43, no. 4: 561–573. 10.1007/BF02293814.

[brb371453-bib-0002] Ballesio, A. , M. R. J. V. Aquino , S. D. Kyle , F. Ferlazzo , and C. Lombardo . 2019. “Executive Functions in Insomnia Disorder: A Systematic Review and Exploratory Meta‐Analysis.” Frontiers in Psychology 10: 101. 10.3389/fpsyg.2019.00101. 30761049 PMC6363670

[brb371453-bib-0003] Baddeley, A. D. , and G. Hitch . 1974. “Working Memory.” In The Psychology of Learning and Motivation: Volume 8, Advances in Research and Theory, edited by G. H. Bower , 47–89. Academic Press.

[brb371453-bib-0004] Barkley, R. A. 1997. ADHD and the Nature of Self‐Control. Guilford Press.

[brb371453-bib-0005] Barkley, R. A. 2010. “Differential Diagnosis of Adults With ADHD: The Role of Executive Function and Self‐Regulation.” Journal of Clinical Psychiatry 71, no. 7: e17. 10.4088/JCP.9066tx1c. 20667287

[brb371453-bib-0006] Barkley, R. A. 2011. Barkley Deficits in Executive Functioning Scale (BDEFS). Guilford Press.

[brb371453-bib-0007] Baroni, M. , M. Scalese , L. Bastiani , S. Cerrai , S. Biagioni , and S. Molinaro . 2025. “Examining the Validity of the Short Problematic Internet Use Test Among Adults and the Link With Impairment of Executive Functions and Well‐Being: A Cross‐Sectional Study.” Current Psychology 44, no. 11: 10026–10039. 10.1007/s12144-025-07824-w.

[brb371453-bib-0008] Bond, T. G. , and C. M. Fox . 2007. Applying the Rasch Model: Fundamental Measurement in the Human Sciences. 2nd ed. Lawrence Erlbaum Associates.

[brb371453-bib-0009] Carrigan, N. , and E. Barkus . 2016. “A Systematic Review of Cognitive Failures in Daily Life: Healthy Populations.” Neuroscience and Biobehavioral Reviews 63: 29–42. 10.1016/j.neubiorev.2016.01.010. 26835660

[brb371453-bib-0010] Cesari, V. , E. Marinari , M. Laurino , A. Gemignani , and D. Menicucci . 2021. “Attention‐Dependent Physiological Correlates in Sleep‐Deprived Young Healthy Humans.” Behavioral Sciences 11, no. 2: 22. 10.3390/bs11020022. 33562527 PMC7915657

[brb371453-bib-0011] Couffe, C. , and G. A. Michael . 2017. “Failures Due to Interruptions or Distractions: A Review and a New Framework.” American Journal of Psychology 130, no. 2: 163–181. 10.5406/amerjpsyc.130.2.0163. 29461714

[brb371453-bib-0012] Cristofori, I. , S. Cohen‐Zimerman , and J. Grafman . 2019. “Executive Functions.” Handbook of Clinical Neurology 163: 197–219. 10.1016/B978-0-12-804281-6.00011-2. 31590731

[brb371453-bib-0013] Diaz‐Marsa, M. , A. Pemau , L. De , et al. 2023. “Executive Dysfunction in Eating Disorders: Relationship With Clinical Features.” Progress in Neuro‐Psychopharmacology and Biological Psychiatry 120: 110649. 10.1016/j.pnpbp.2022.110649. 36181959

[brb371453-bib-0014] Dorans, N. J. , and P. W. Holland . 1993. “DIF Detection and Description: Mantel‐Haenszel and Standardization.” In Differential Item Functioning, edited by P. W. Holland and H. Wainer , 35–66. Lawrence Erlbaum Associates.

[brb371453-bib-0015] Fogel, Y. , Y. Gilboa , and S. Meyer . 2024. “Exploratory Factor Analysis and Convergent Validity of the Adult Executive Function Inventory (ADEXI).” American Journal of Occupational Therapy 78, no. 6: 7806205140. 10.5014/ajot.2024.050686. 39405580

[brb371453-bib-0016] García‐Villamisar, D. , M. Jodra , G. P. Sáez‐Suanes , and L. B. Thorell . 2020. “Spanish Version of the Adult Executive Functioning Inventory (ADEXI): Psychometric Properties in Adults With Autism Spectrum Disorders and Intellectual Disability.” Journal of Psychopathology and Clinical Psychology 25, no. 2: 121–129. 10.5944/RPPC.26183.

[brb371453-bib-0017] Groves, N. B. , E. L. Wells , E. F. Soto , et al. 2022. “Executive Functioning and Emotion Regulation in Children With and Without ADHD.” Research on Child and Adolescent Psychopathology 50, no. 6: 721–735. 10.1007/s10802-021-00883-0. 34762251 PMC9091051

[brb371453-bib-0018] Gulledge, C. M. , V. A. Lizzio , D. G. Smith , E. Guo , and E. C. Makhni . 2020. “What Are the Floor and Ceiling Effects of Patient‐Reported Outcomes Measurement Information System Computer Adaptive Test Domains in Orthopaedic Patients? A Systematic Review.” Arthroscopy: The Journal of Arthroscopic & Related Surgery 36, no. 3: 901–912.e7. 10.1016/j.arthro.2019.09.022. 31919023

[brb371453-bib-0019] Hancock, M. , J. L. Tapscott , and P. N. Hoaken . 2010. “Role of Executive Dysfunction in Predicting Frequency and Severity of Violence.” Aggressive Behavior 36, no. 5: 338–349. 10.1002/ab.20353. 20593426

[brb371453-bib-0020] Herrera, A.‐N. , and J. Gómez . 2008. “Influence of Equal or Unequal Comparison Group Sample Sizes on the Detection of Differential Item Functioning Using the Mantel‐Haenszel and Logistic Regression Techniques.” Quality & Quantity: International Journal of Methodology 42, no. 6: 739–755. 10.1007/s11135-006-9065-z.

[brb371453-bib-0021] Holst, Y. , and L. B. Thorell . 2018. “Adult Executive Functioning Inventory (ADEXI): Validity, Reliability, and Relations to ADHD.” International Journal of Methods in Psychiatric Research 27, no. 1: e1567. 10.1002/mpr.1567. 28497641 PMC6877129

[brb371453-bib-0022] Latkin, C. A. , C. Edwards , M. A. Davey‐Rothwell , and K. E. Tobin . 2017. “The Relationship Between Social Desirability Bias and Self‐Reports of Health, Substance Use, and Social Network Factors Among Urban Substance Users in Baltimore, Maryland.” Addictive Behaviors 73: 133–136. 10.1016/j.addbeh.2017.05.005. 28511097 PMC5519338

[brb371453-bib-0023] Lezak, M. D. , D. B. Howieson , E. D. Bigler , and D. Tranel . 2012. Neuropsychological Assessment. 5th ed. Oxford University Press.

[brb371453-bib-0024] Lima, I. M. M. , A. D. Peckham , and S. L. Johnson . 2018. “Cognitive Deficits in Bipolar Disorders: Implications for Emotion.” Clinical Psychology Review 59: 126–136. 10.1016/j.cpr.2017.11.006. 29195773 PMC6404979

[brb371453-bib-0025] Linacre, J. M. 2002a. “Optimizing Rating Scale Category Effectiveness.” Journal of Applied Measurement 3, no. 1: 85–106. 11997586

[brb371453-bib-0026] Linacre, J. M. 2002b. “What Do Infit and Outfit, Mean‐Square and Standardized Mean?” Rasch Measurement Transactions 16, no. 2: 878.

[brb371453-bib-0027] Linacre, J. M. 2026. A User's Guide to Winsteps Ministep Rasch‐Model Computer Programs, Program Manual 5.11.0 . Winsteps.com. https://www.winsteps.com/a/Winsteps‐Manual.pdf.

[brb371453-bib-0028] Martinková, P. , A. Drabinová , Y. L. Liaw , E. A. Sanders , J. L. McFarland , and R. M. Price . 2017. “Checking Equity: Why Differential Item Functioning Analysis Should be a Routine Part of Developing Conceptual Assessments.” CBE Life Sciences Education 16, no. 2: rm2. 10.1187/cbe.16-10-0307. 28572182 PMC5459266

[brb371453-bib-0029] Masters, G. N. (2019). Partial credit model. In W. J. van der Linden (Ed.), Handbook of item response theory (Vol. 1, pp. 109–126). Chapman and Hall/CRC.

[brb371453-bib-0030] Niedźwieńska, A. , J. Sołga , P. Zagaja , and M. Żołnierz . 2020. “Everyday Memory Failures Across Adulthood: Implications for the Age Prospective Memory Paradox.” PLoS ONE 15, no. 9: e0239581. 10.1371/journal.pone.0239581. 32976533 PMC7518607

[brb371453-bib-0031] Rock, P. L. , J. P. Roiser , W. J. Riedel , and A. D. Blackwell . 2014. “Cognitive Impairment in Depression: A Systematic Review and Meta‐Analysis.” Psychological Medicine 44, no. 10: 2029–2040. 10.1017/S0033291713002535. 24168753

[brb371453-bib-0032] Roth, R. M. , P. K. Isquith , and G. A. Gioia . 2005. Behavior Rating Inventory of Executive Function—Adult Version: Professional Manual . Psychological Assessment Resources.

[brb371453-bib-0033] Salehinejad, M. A. , E. Ghanavati , M. H. A. Rashid , and M. A. Nitsche . 2021. “Hot and Cold Executive Functions in the Brain: A Prefrontal‐Cingular Network.” Brain and Neuroscience Advances 5: 23982128211007769. 10.1177/23982128211007769. 33997292 PMC8076773

[brb371453-bib-0034] Siman‐Tov, M. , I. Radomislensky , N. Knoller , et al. 2016. “Incidence and Injury Characteristics of Traumatic Brain Injury: Comparison Between Children, Adults and Seniors in Israel.” Brain Injury 30: 83–89. 10.3109/02699052.2015.1104551. 26734841

[brb371453-bib-0035] Sousa, V. D. , and W. Rojjanasrirat . 2011. “Translation, Adaptation and Validation of Instruments or Scales for Use in Cross‐Cultural Health Care Research: A Clear and User‐Friendly Guideline.” Journal of Evaluation in Clinical Practice 17, no. 2: 268–274. 10.21203/rs.3.rs-4001736/v1. 20874835

[brb371453-bib-0036] Tennant, A. , and P. G. Conaghan . 2007. “The Rasch Measurement Model in Rheumatology: What Is It and Why Use It? When Should It be Applied, and What Should One Look for in a Rasch Paper?” Arthritis and Rheumatism 57, no. 8: 1358–1362. 10.1002/art.23108. 18050173

[brb371453-bib-0037] Townes, P. , C. Liu , P. Panesar , et al. 2023. “Do ASD and ADHD Have Distinct Executive Function Deficits? A Systematic Review and Meta‐Analysis of Direct Comparison Studies.” Journal of Attention Disorders 27, no. 14: 1571–1582. 10.1177/10870547231190494. 37565325 PMC10637091

[brb371453-bib-0038] Welsh, M. C. , and B. F. Pennington . 1988. “Assessing Frontal Lobe Functioning in Children: Views From Developmental Psychology.” Developmental Neuropsychology 4, no. 3: 199–230. 10.1080/87565648809540405.

[brb371453-bib-0039] Wilson, B. A. , N. Alderman , P. W. Burgess , H. C. Emslie , and J. J. Evans . 1996. Behavioral Assessment of Dysexecutive Syndrome Manual. Thames Valley Test Company.

[brb371453-bib-0040] Zainal, N. H. , and M. G. Newman . 2022. “Executive Functioning Constructs in Anxiety, Obsessive‐Compulsive, Post‐Traumatic Stress, and Related Disorders.” Current Psychiatry Reports 24, no. 12: 871–880. 10.1007/s11920-022-01390-9. 36401677 PMC9676877

[brb371453-bib-0041] Zwick, R. , D. T. Thayer , and C. Lewis . 1999. “An Empirical Bayes Approach to Mantel‐Haenszel DIF Analysis.” Journal of Educational Measurement 36, no. 1: 1–28. 10.1111/j.1745-3984.1999.tb00543.x.

